# The Cure of Stammering

**Published:** 1894-05-05

**Authors:** 


### Studies in Applied Sociology.

### VIII.—THE CURE OF STAMMERING.

It will be remembered how Gwynplaine, "L'Homme que Rit," of Victor Hugo's novel, suffered from the barbarous mangling of his face to make it resemble a laughing mask, chiefly in that it made everyone see cause for mirth in everything he said. No matter how serious his theme, how fervid his words, his listeners looked at his face and thought he was joking. The fate of the stammerer is akin to this. He, too, no matter how much in earnest he may be, is regarded only as a Dundrearyish joke. Yet the impediment is cruelly distressing to the sufferer, who finds speech fail him when he needs it most. No doubt nervousness, produced by the consciousness that he will stammer when he speaks, helps to make the difficulty worse; but mere nervousness—using the word in its popular sense of self-consciousness—will not account for the impediment itself.

Nevertheless, much may be done in the nursery to prevent the acquisition of correct habits of speech. Children naturally lisp, transform *r* into *l*, *th* into *v*, and the like, and parents, amused at the baby tongue, answer in the same style. Thus the faulty habit, instead of being cured, becomes confirmed. Even with children who stammer, parents, by way of a joke, put on a stammer in speaking to the child, and this encourages the defect. The evil habit once acquired becomes more marked as the child reaches the age of self-consciousness, and knows his liability to stammer. It is in this way that an impediment, which, when the stammerer is off his guard is hardly apparent, or disappears entirely, makes him almost inarticulate when excited or deeply in earnest. To cure this it is necessary to undergo a thorough course of voice-training, in which breathing and articulation will each have due attention. It must be remembered, however, that voice-training alone will not cure every case. When stammering is merely a symptom of epilepsy or hysteria, the doctor must be consulted as well as the elocutionist, and medical or surgical treatment is a necessary prelude to any improvement of speech. This was always strongly insisted on by the late Emile Behnke, whose method, which is now carried on by his wife and daughter, has had the most successful results, even in cases of confirmed stammering. It is comprehensible that any diseased condition of nose or throat will very probably affect the speech, but other and less obvious causes, which bring about neurosis, may be equally serious in their results.

Many people regard stammering as merely "a bad habit." In so far as the defect becomes more marked in conditions of excitement and self-consciousness this is, after a fashion, true; and the mind may be brought in to help the removal of the impediment. There is a story told of a woman in Strasburg, who was told that the stammering with which she was afflicted was caused by the devil. Therefore, every time she felt that the impediment was about to interfere with her speech she muttered under her breath, "In the name of the Father, the Son, and the Holy Ghost," and then turned round, spat, and said to the devil, "That is for thee!" This exorcism, it is said, always stopped the attack. Kingsley, who himself had conquered the trouble, advised stammerers when speaking to accompany each syllable with some movement of hand or foot, in order by the muscular effort to distract attention from the speaking. This is helpful in cases where the stammering arises from timidity, shyness, or anxiety, but will not prove effective in all.

The first essential in the cure of stammering is correct breathing, because one of the habits of confirmed stammerers is to speak with exhausted lungs Mr. Behnke's method commenced with what he called "diaphragmatic drill"—that is, breathing from the diaphragm instead of the short, jerky, hard, clavicular breathing which is common. Diaphragmatic breathing, where the resistance is only of soft parts, not only permits of the full aeration of the lung, but can be controlled to a nicety, whereas clavicular breathing, pushing against the harder parts of the thorax, is apt to be short and jerky. This mere matter of proper breathing will often enable a patient to pronounce the letters over which he habitually stumbled. After the breathing comes "phonation drill," *i.e.*, the proper use of the vocal chords. Why is it that while a trained orator can address a large meeting for an hour or two on end without fatigue, and without apparently raising his voice above the ordinary tone of conversation his untrained rival shouts and shrieks himself into a state of husky exhaustion in less than half the time? Because the first knows how to use his voice. He pronounces each syllable clearly, yet not with "barn-storming" emphasis; he knows how to produce a clear and resonant tone, and that without straining or forcing his voice. What the ordinary man needs, the stammerer needs still more. Above all, he needs practice in that prompt and sure action called "shock of the glottis." To attain this the patient should be told to sing a number of *staccato* tones, each one preceded by a short inspiration. The throat thus becomes accustomed to prompt, sharp action, and the voice is more easily managed.

It is an old joke that stammerers can sing when they cannot speak. This is because in singing the whole stress is laid on the vowels, while the consonants are passed over lightly. The reverse of this occurs in speaking, where, especially in our English tongue, the vowels are all but ignored, and the whole emphasis falls on the consonants. But it is over the consonants that the stammerer falls. Therefore for him the rule must be "take care of your vowels and your consonants will take care of themselves"—as indeed they will, and fall into their proper places without effort in the end. Mr. Behnke also recommends speaking slowly and distinctly with teeth overlapping and held firmly together. The exercise is not easy even for those without impediment, and the stammerer who can manage it need not thereafter be at much anxiety as to speaking in the natural manner. He will find it no such difficult task. Though it will be well for him always to speak slowly, with distinct articulation, and to maintain a good level of general health, in the absence of which one's weak points are always likely to come to the surface.

